# Habitual Khat and Concurrent Khat and Tobacco Use Are Associated With Subjective Sleep Quality

**DOI:** 10.5888/pcd11.130234

**Published:** 2014-05-22

**Authors:** Motohiro Nakajima, Anisa Dokam, Abed Naji Kasim, Mohammed Alsoofi, Najat Sayem Khalil, Mustafa al’Absi

**Affiliations:** Author Affiliations: Motohiro Nakajima, University of Minnesota Medical School, Duluth, Minnesota; Anisa Dokam, Mohammed Alsoofi, Taiz University, Taiz, Yemen; Abed Naji Kasim, Najat Sayem Khalil, Sana’a University, Sana’a, Yemen.

## Abstract

**Introduction:**

Khat (*Catha edulis*) is widely used in East Africa and the Middle East, often in combination with tobacco smoking. Sleep disturbance has been linked with habitual khat use; however, no systematic attempt has been made to test the hypothesis that use of khat and khat and tobacco in combination are related to sleep disturbance. Sleep disturbances are associated with dysregulations in emotional and physiological functions and can increase health risks.

**Methods:**

We developed and used the Arabic version of the Pittsburgh Sleep Quality Index (PSQI) to conduct a cross-sectional study in Yemen examining subjective sleep quality in 151 concurrent users of khat and tobacco, 141 khat-only users, and 92 nonusers. Measures on subjective mood were also collected. A series of analyses of variance and χ^2^ tests were conducted to test whether khat and tobacco use was linked with sleep disturbances.

**Results:**

Concurrent users of tobacco and khat and khat-only users showed greater sleep disturbances than nonusers as assessed by the PSQI global scores (all *P* values < .001) and component scores. PSQI scores were correlated with negative and positive mood (all *P* values < .004).

**Conclusion:**

Sleep disturbances may be 1 mechanism of the link between khat, tobacco, and negative health outcomes. Our findings may be useful in developing targeted prevention and harm-reduction strategies to minimize health care burdens associated with these substances. Our study also provides initial support for the Arabic version of PSQI.

## Introduction

Khat use (*Catha edulis*) is widely accepted in East Africa and the Middle East and is associated with increased risk for physical diseases and mental disorders (1). The main constituent of khat is cathinone whose chemical structure is similar to that of amphetamine (2,3). Cathinone stimulates dopaminergic pathways (2) that are involved in regulation of sleep and awake time (4). Users report positive experiences while chewing khat (1,5); however, these are followed by negative symptoms including anxiety, depression, and insomnia (1,6–8). Epidemiological data show that from 30% to 70% of the population of Ethiopia, Kenya, and Yemen chew khat (9–11). Khat is also used in Europe by immigrants from East Africa and the Middle East (6,12). Khat plays an important role in socialization (1) and is often used with tobacco (people smoke tobacco while chewing khat) (6,12,13).

Khat users report sleep disturbances; however, data showing an association between the two are limited (6,8,10). Only 1 study using a validated tool reported an association between sleep disturbances and use of tobacco and khat (14). Furthermore, no study has tested this association in concurrent users of khat and tobacco. Studies are needed to clarify the association between sleep disturbances, khat use, and tobacco use because long-term insomnia and habitual khat and tobacco use are independent risk factors for physical and psychological disorders (15–18). Such studies would expand understanding of the underlying mechanisms of khat and tobacco use and their association with illness and death, which could have important public health implications in countries where khat and khat and tobacco combined are widely used (13,19). We hypothesized that habitual concurrent users of khat and tobacco would report worse subjective sleep quality than nonusers.

We used the Arabic version of the Pittsburgh Sleep Quality Index (PSQI) (20) to examine whether khat and tobacco use were associated with sleep disturbances.

## Methods

### Participants

Our study used a cross-sectional method. Participants were recruited at Taiz and Sana’a universities in Yemen through flyer postings around the campus and in the community. Data collection was completed in 2012 over a period of 2 months. To be eligible, participants needed to be free from any current or recent history of major physical and psychiatric diseases or of taking prescribed medications. A total of 401 participants completed the study. The sample included 151 people who defined themselves as concurrent users of khat and tobacco (77 men and 74 women), 141 who defined themselves as khat-only users (65 men and 76 women), and 92 nonusers (40 men and 52 women) defined as those who were not currently using khat or tobacco and had never used these substances daily. Seventy-eight percent of participants were recruited in Taiz. The proportions of concurrent users, khat-only users, and nonusers were comparable across the 2 sites (*P* = .15). Seventeen participants who identified themselves as tobacco-only users (15 men and 2 women) were excluded from the current analysis because of the small sample size, which led to the final sample of 384. All participants read and signed a consent form approved by the research ethical committees at Taiz or Sana'a universities.

### Apparatus and procedure

The Pittsburgh Sleep Quality Index (PSQI) (20) consists of 19 questions that evaluate qualitative and quantitative dimensions of sleep disturbances. These items are calculated into 7 component scores: sleep quality, sleep latency, sleep duration, habitual sleep efficiency, sleep disturbances, use of sleep medication, and daytime dysfunction (20). Each component score ranges from 0 (good quality) to 3 (poor quality). The sum of these 7 component scores yields the global PSQI score, with the highest score of 21 indicating worst subjective sleep quality. The authors of the PSQI also suggested a cut off of less than 5 on the global score to classify “poor” sleeper from “good” sleeper (20). Internal consistency reported by the original authors was 0.83 (20). Good PSQI test–retest reliability has been reported (20), and the scale has shown high validity in clinical and healthy populations (20–23). The PSQI has been translated into different languages and used in studies that reported adequate psychometric properties (22–24). For our study, we developed an Arabic version of the PSQI with the permission of the PSQI authors. We translated the original English version into Arabic and then back-translated into English.

We also used a subjective mood questionnaire (25), which asked participants to describe themselves as relaxed, depressed, sad, discontented, afraid, angry, anxious, or hungry. Each of these items included an 8-point scale that asked participants to rate the feeling on a scale of 1 to 8, with 1 being “not at all” and 8 being “extremely.” Participants were asked to rate their feelings during the past week. Our preliminary analysis showed that reliability was highest when only negative mood items (ie, depressed, sad, discontented, afraid, angry, and anxious) were included (Cronbach’s α = .79). Therefore, mean scores of these 6 items were combined into an index hereafter referred to as “negative mood” and used with ratings of “relaxed,” and “hungry” as subjective mood measures in the analysis. In addition, we collected demographic information such as age and years of education.

### Data analysis

Analyses of covariance (ANCOVAs) were conducted on a series of 3 groups (concurrent users of khat and tobacco, khat-only users, nonusers) each divided into 2 groups by sex for a total of 6 groups to determine the extent to which khat and tobacco use were associated with subjective sleep quality. A sex variable was included as an independent factor, because prior work reported its relationships with sleep patterns and sleep problems (20,26). Site was adjusted in these models because the primary purpose of data collection from 2 sites, Taiz and Sana’a universities, was to maximize the sample size rather than to test site differences. To assess internal consistency of the global PSQI scores, Cronbach's α was calculated by using the 7 component scores. The construct validity of the scale was assessed by correlational analysis conducted between the global PSQI scores and subjective mood measures (ie, negative mood, relaxed, and hungry) and ANCOVAs described above. Demographic variables were analyzed by a series of analyses of variance and χ^2^ tests for the 6 groups. Bonferroni correction was applied for post-hoc multiple comparisons. *P* values less than .05 were considered significant. SPSS version 19 (IBM Corp, Armonk, New York) was used for data analysis. We note that reported results varied in degrees of freedom because of occasional missing responses.

## Results

### Participant characteristics

On average, concurrent users of tobacco and khat and khat-only users reported that they chewed khat 5 hours a day (standard deviation [SD], 2.3) 5 times (SD, 2.3) a week. Mean age of onset of khat use was 17 years (SD, 5.8), and the rate of daily use was 62%. Concurrent users reported smoking an average of 17 cigarettes (SD, 11.7) and 1 shisha (waterpipe) (SD, 0.6) per day. Approximately 40% were daily smokers. A significant group-by-sex interaction in age (*F*_2,378_ = 12.7, *P* < .001) indicated that women were older than men in the khat-only group (*P* = .01), and men were older than women in the nonusers group (*P* < .001) (Table). This difference was not observed in concurrent users (*P* = .68). There was a significant group–sex interaction in years of education (*F*_2,340_ = 3.50, *P* = .03), indicating longer education in men than in women among concurrent and khat-only users (all *P* values < .003). This sex difference was not found in nonusers (*P* = .65). The rate of being married was lower in female nonusers relative to other groups (χ^2^ = 23.7, *P* = .001), which was consistent with age distribution. The rate of employment was greater in men than in women. (χ^2^ = 48.7, *P* < .001). Preliminary analysis found correlations between global PSQI scores and age (*r*_384_ = .12, *P* = .02), years of education (*r*_346_ = −.15, *P* = .005), and employment status (*r*_384_ = .11, *P* = .04). Thus, these variables were included as covariates in the subsequent ANCOVAs on the global scores and the component scores.

**Table:** Sample Characteristics and Sleep Disturbance Scores of Nonusers, Khat-Only Users, and Concurrent Khat and Tobacco Users

Characteristic	User Group					
	Nonusers		Khat-Only Users		Concurrent Users of Khat and Tobacco	
	Men (n = 40)	Women (n = 52)	Men (n = 65)	Women (n = 76)	Men (n = 77)	Women (n = 74)
**Demographics**						
Age, y, mean (SE)^a^	32.9 (1.5)	24.3 (1.3)	28.1 (1.2)	31.8 (1.1)	30.8 (1.1)	31.5 (1.1)
Education, y, mean (SE)^a^^,^ b ^,^ c	15.7 (0.7)	15.3 (0.6)	15.7 (0.5)	12.4 (0.5)	14.7 (0.5)	12.3 (0.5)
Married, %^d^	60.0	17.3	58.5	63.2	48.1	60.8
Employed, %^e^	77.5	30.8	60.0	23.7	54.5	25.7
**Sleep disturbance, overall PSQI score^f^**						
Global score, mean (SE)^b^^,^ c	2.3 (0.5)	4.5 (0.4)	5.7 (0.4)	6.3 (0.4)	5.8 (0.4)	7.1 (0.4)
Good sleeper, %^d^	90.0	63.5	49.2	42.1	45.5	40.5
**PSQI component scores, mean (SE)^g^**						
Sleep quality^b^	0.4 (0.1)	0.5 (0.1)	0.9 (0.1)	0.9 (0.1)	1.0 (0.1)	1.1 (0.1)
Sleep latency^a^^,^ b	0.5 (0.2)	1.2 (0.2)	1.7 (0.1)	1.5 (0.1)	1.8 (0.1)	1.7 (0.1)
b	0.3 (0.1)	0.4 (0.1)	0.6 (0.1)	0.6 (0.1)	0.5 (0.1)	0.7 (0.1)
Habitual sleep efficiency^b^^,^ c	0.2 (0.1)	0.4 (0.1)	0.4 (0.1)	0.5 (0.1)	0.5 (0.1)	1.0 (0.1)
Sleep disturbances^b^^,^ c	0.7 (0.1)	1.2 (0.1)	1.3 (0.1)	1.4 (0.1)	1.3 (0.1)	1.5 (0.1)
Use of sleep medication	0.1 (0.1)	0.1 (0.1)	0.1 (0.1)	0.2 (0.1)	0.1 (0.1)	0.3 (0.1)
Daytime dysfunction^b^^,^ c	0.1 (0.1)	0.6 (0.1)	0.7 (0.1)	1.2 (0.1)	0.6 (0.1)	0.9 (0.1)

Abbreviations: SE, standard error; PSQI, Pittsburgh Sleep Quality Index (20).

a Interaction between sex and user status was significant (*P* <.05).

b Main effect of user group was significant (*P* <.05).

c Main effect of sex was significant (*P* <.05).

d User group was associated with characteristic (*P* <.05).

e Sex was associated with employment status (*P* <.05).

f Global PSQI scores ranged from 0 to 21. Percentage of “good sleepers” was calculated based on a cutoff score of 5 in the global PSQI scores.

g PSQI component scores ranged from 0 to 3.

### Psychometric properties of the PSQI

**Figure 1:**
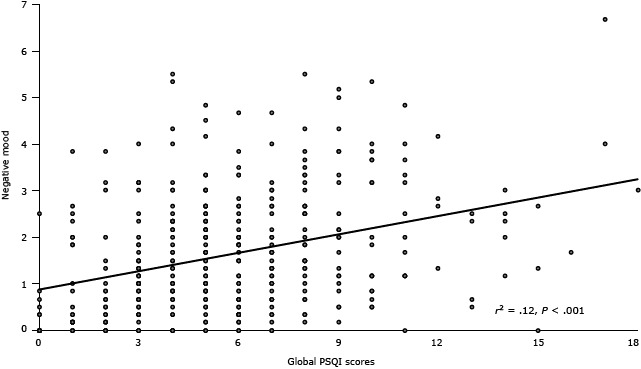
Association between sleep disturbances as measured by the Pittsburgh Sleep Quality Index (20) and negative mood as measured by a subjective mood questionnaire (25). Abbreviation: PSQI, Pittsburgh Sleep Quality Index.

The Cronbach’s α of PSQI in the entire sample was .66. When conducted in each group, the internal consistency was .67 in concurrent users, .57 in khat-only users, and .61 in nonusers. Component-to-global score correlations ranged between .50 (sleep quality) and .22 (use of sleep medications). Correlational analysis conducted to test construct validity revealed that participants who reported having a greater number of sleep problems (greater total PSQI scores) were more likely to have enhanced negative mood (*r*_384_ = .35, *P* < .001 (Figure 1) and decreased positive mood as assessed by “relaxed” (*r*_384_ = −.15, *P* = .003). Multiple regression analysis further revealed that the relationship between negative mood and sleep disturbances remained significant after controlling for group and sex (β = .27, *t* = 5.70, *P* < .001). Sleep disturbances were not associated with “hungry” (*r*_384_ = .03, *P* = .52).

Negative mood was positively correlated with all sleep component scores except sleep duration (all *r*_384_ > .11, all *P* values < .03). Positive mood was negatively associated with sleep quality and sleep disturbances (all *r*_384_ > −.15, all *P* values < .003). Hunger was positively related to sleep disturbances (all *r*_384_ > .13, all *P* values = .01). The linkages of negative mood with sleep quality, sleep latency, sleep disturbances, use of sleep medication, and daytime dysfunction remained significant after adjusting for group and sex (all β > .11, all *t*-statistics > 2.17, all *P* values < .04). Also, the findings on positive mood remained significant after taking into account group and sex influences (all β > −.09, all t-statistics > 1.98, all *P* values < .05).

### Associations between khat and tobacco use and sleep quality

**Figure 2:**
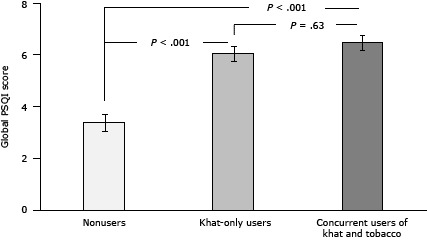

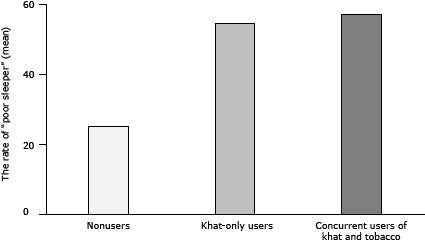
Differences in subjective sleep quality as a function of khat and tobacco use status. Sleep quality measured using the Pittsburgh Sleep Quality Index (PSQI) (20). In Figure 2a, values are the mean, and the I-beam lines in each bar indicate standard error of the mean. In Figure 2b, entries show percentages of participants classified as poor sleepers as determined by global PSQI scores greater than 5. Abbreviation: PSQI, Pittsburgh Sleep Quality Index. **Table:** 

The global PSQI scores were greater (poorer sleep quality) in concurrent khat and tobacco users and khat-only groups relative to nonusers (main effect of group: *F*_2,377_ = 29.8, *P* < .001; multiple comparisons: all *P* values < .001), and the difference between the 2 khat groups was comparable (*P* = .63 [Figure 2a]). Women had higher scores than men (*F*_1,377_ = 16.6, *P* < .001). A model with additional covariates (ie, age, education, and employment status) further revealed a significant group–sex interaction (*F*_2,336_ = 3.33, *P* = .04). ANCOVAs conducted in each group found more sleep problems in women than in men among nonusers (*F*_1,85_ = 11.4, *P* = .001), but this sex difference was not observed among the 2 khat groups (all *P* values > .10). A χ^2^ analysis examining associations of khat and tobacco use with “good” versus “poor” sleeper (based on a cutoff of >5 in the global PSQI scores) found that the number of nonusers categorized as poor sleepers was smaller than that of concurrent and khat-only users (χ^2^ = 26.8, *P* < .001 [Figure 2b]).

Characteristic	Nonusers	Khat-Only Users	Concurrent Users of Khat and Tobacco
Global PSQI score, mean (standard error of the mean)	3.38 (0.33)	5.99 (0.29)	6.45 (0.27)
Poor sleepers, %	25.0	54.6	57.0

*P* < .001 for difference between nonusers and khat-only users. *P* < .001 for difference between nonusers and concurrent users of khat and tobacco. *P* < .63 for difference between khat-only users and concurrent users of khat and tobacco.

Regarding component scores, sleep quality (*F*_2,377_ = 22.9, *P* < .001), sleep latency (*F*_2,377_ = 19.2, *P* < .001), sleep disturbances (*F*_2,377_ = 16.8, *P* < .001), and daytime dysfunction (*F*_2, 377_ = 12.7, *P* < .001) were greater (worse) in concurrent users and khat-only users than in nonusers (post-hoc tests, all *P* values < .002); the 2 khat groups did not differ in these components (Table). The finding of sleep latency was further qualified by a significant group–sex interaction (*F*_2,377_ = 6.31, *P* = .002) indicating comparable scores between men and women among the khat and concurrent khat and tobacco users groups (all *P* values > .28), and greater scores were found in women than in men among nonusers (*F*_1,89_ = 10.8, *P* = .001). Sleep duration (*F*_2,377_ = 3.28, *P* = .04) and habitual sleep efficiency (*F*_2,377_ = 6.20, *P* = .002) were greater in concurrent users than in nonusers (post-hoc tests, all *P* values < .05). Relative to men, women had greater sleep disturbances (*F*_1,377_ = 20.8, *P* < .001), daytime dysfunction (*F*_1,377_ = 25.4, *P* < .001) and habitual sleep efficiency (*F*_1,377_ = 7.16, *P* = .008). Medication use did not differ by group or by sex (all *P* values > .10).

Similar patterns of results were observed when additional covariates were included in the model. Significant group differences (all values, *F*_2, 336_ > 11.6, all *P* values < .001) with greater scores in concurrent and khat-only users relative to nonusers were found in sleep quality, sleep latency, sleep disturbances, and daytime dysfunction (all *P* values < .009). The findings of sleep disturbances and sleep latency were further qualified by significant group–sex interactions (all values, *F*_2,336_ > 4.19, all *P* values < .02) reflecting no sex differences among the 2 khat groups and greater scores in women than in men among nonusers. Concurrent users had greater scores than nonusers in habitual sleep efficiency (*F*_2,336_ = 4.14, *P* = .02; post-hoc tests: all *P* values < .02). Women had greater scores in sleep disturbances and daytime dysfunction (all values, *F*_1,336_ > 12.6, all *P* values <.001). No group or sex difference was found in medication use (all *P* values > .35).

A series of correlational analyses found weak but significant positive associations of the global PSQI scores with reported times of khat-chewing per week (*r*_291_ = .12, *P* = .04) and number of cigarettes smoked per day (*r*_88_ = .21, *P* = .05).

## Discussion

Our study was among the first to demonstrate the association between impaired subjective sleep quality and habitual use of khat and khat and tobacco combined among an adult sample. Concurrent users of khat and tobacco as well as khat-only users showed greater PSQI scores than nonusers. Fewer than half of participants in the 2 khat groups were classified as good sleepers whereas most nonusers were in the good-sleeper category. Results of the PSQI component scores yielded similar patterns: concurrent and khat-only users had worse levels of sleep quality, sleep latency, sleep disturbances, and daytime dysfunction than nonusers. These findings confirm our hypothesis about sleep disturbances in habitual khat users and concurrent khat and tobacco users and are consistent with findings from prior studies of khat chewers (6,8,10,14).

Findings of our study have clinical and public health implications. For example, in countries where khat use is legal, health care professionals could provide khat users and concurrent khat and tobacco users with detailed information on how these substances affect health behaviors such as sleep and how they increase physical and mental health risks. Also, our data are beneficial in providing proper education to vulnerable people to protect them from additional burdens to their life. This is a serious concern among immigrant populations, because they chew khat to manage stress and maintain cultural identity during the period of acculturation. More research is needed to elucidate social and psychological determinants that mediate the link between khat use and combined khat and tobacco use and illness. Such work is essential in developing effective programs to reduce public health burdens associated with these substances.

Our study found preliminary evidence for a dose-dependent relationship between khat use and subjective sleep quality. We observed weak but significant positive correlations between reported frequency of khat sessions per week and sleep disturbances. This finding and similar reports from other studies (14) suggest clinical implications, because accumulating evidence indicates linkages of excessive khat use (1,7,17) and long-term insomnia (15,16) with mental and physical diseases. These observations suggest a bidirectional relationship between substance abuse and sleep disturbances, which may increase risk for the development of chronic diseases and drug addiction. However, this hypothesis has not been examined.

In general, our study did not find differences in subjective sleep quality between concurrent khat and tobacco users and khat-only users, suggesting that the influence on sleep quality was driven primarily by khat use. Research has reported associations between insomnia and khat use (6,8,10,14) and insomnia and tobacco use (14). However, no study has examined sleep difficulties in concurrent users of these substances. Although we note a lack of tobacco-only users (Nakajima et al [13] describe potential cultural influences related to the absence of this group) and a lack of detailed assessment of substance use history (duration, dependence), it is possible that there is no additive effects of khat and tobacco use on sleep disorders.

The reliability of the Arabic PSQI was lower than the original (20) and translated versions (22). In contrast, when examined in each group, internal consistency in nonusers was comparable to that of healthy individuals in Japanese (22), Persian (23), and Hebrew (24) samples. The PSQI was developed to assess subjective sleep quality in psychiatric clinical populations. Therefore, some items in the questionnaire may not appropriately capture sleep-related problems among nonclinical populations. Cultural factors may also be associated with the results. For example, in our study the correlation between the “use of sleep medication” component score and the global scores was low. The extent to which taking sleep medication is accepted in Middle Eastern society is not clear. Possibly, some questions in the scale did not capture sociocultural influences on patterns of sleep.

The Arabic PSQI showed high construct validity. Concurrent users of khat and tobacco and khat-only users had greater global scores and greater scores on components of sleep quality, sleep latency, sleep disturbances, and daytime dysfunction than scores for nonusers, indicating the ability of the scale to discriminate khat and tobacco users from nonusing controls in terms of subjective sleep quality. In addition, the higher global scores as well as component scores were associated with greater levels of negative mood, findings that support prior work (21). Although modest internal consistency calls for a cautious interpretation, the findings of group differences and correlations provide initial support for the usefulness of Arabic PSQI, the global score, and the component scores in assessing subjective quality and quantity of sleep among khat chewers and tobacco smokers.

This study was novel in terms of examining sex differences in subjective sleep quality among adults in Yemen. The current finding that women in general were more likely to report sleep disturbances than men was consistent with a study that used a meta-analysis (27). Hormonal changes due to menstrual cycle, pregnancy, and menopause are associated with sleep (26). Also, recent studies have shown the role of the menstrual phase in substance use, such as craving for cigarettes (28). We found some indications of moderating effects of khat and tobacco use in sex differences in subjective sleep quality. Global PSQI (after adjusting for covariates) and sleep latency scores were higher (worse) in women than in men among nonusers only. Chronic use of substances such as tobacco and khat may be linked with impairment of sleep quality, particularly among men, resulting in reduction of differences attributable to sex. More research is needed to identify biological and psychosocial determinants that would predict substance use and sleep disturbance in men and women.

The findings of our study are limited primarily by the use of a cross-sectional design. It is not clear whether habitual khat and tobacco use diminishes sleep quality or if people who are predisposed to have sleep problems chew khat and smoke tobacco. Future research should develop a cohort group and conduct longitudinal assessments to identify the type of person who is at risk for initiating khat and tobacco use. Psychosocial measures should also be assessed to account for comorbidity. Inclusion of objective measures such as polysomnography may be useful in elucidating mechanisms of khat and tobacco use and sleep quality. Nevertheless, our study was among the first to systematically assess subjective sleep quality among khat chewers and tobacco smokers in the Middle East.

Our study found that concurrent users of khat and tobacco and khat-only users reported greater sleep disturbances than nonusers. The Arabic version of the PSQI had an adequate reliability and construct validity. More research to identify mechanisms responsible for khat and tobacco use and illness is critical in improving public health in countries where use of these substances is widely accepted.
